# String of successful trials in SLE: have we cracked the code?

**DOI:** 10.1136/lupus-2019-000380

**Published:** 2020-01-02

**Authors:** Ronald van Vollenhoven

**Affiliations:** 1 Amsterdam University Medical Center, Amsterdam, The Netherlands; 2 Department of Rheumatology, Amsterdam Rheumatology Center, Amsterdam, The Netherlands

**Keywords:** systemic lupus erythematosus, disease activity, treatment

As the year 2019 draws to a close, I am reminded how 30 years ago, almost without a warning, the communist dictatorships in Eastern Europe started falling, one after the other. This fall, we are seeing another unlikely and largely unexpected but hoped for grouping of events: a series of successful phase III trials in SLE. These successes follow on a longer period during which successes in smaller, phase II trials were emerging with a range of drugs, including ustekinumab,^1^ baricitinib,^2^ cenerimod^3^ and others. But now, in short succession, three large phase III trials meeting their primary outcome of efficacy were published or announced (table 1).

**Table 1:** The recent string of successful phase III trials in SLE

Trial	Drug	Patients	Primary outcome	
TULIP 2	Anifrolumab	General SLE	BICLA (at 52 weeks)	47.8% versus 31.5%
AURORA	Voclosporin	Lupus nephritis	Renal response (at 52 weeks)	40.8% versus 22.5%
BLISS-LN	Belimumab	Lupus nephritis	Primary efficacy renal response over 2 years	43% versus 32%

*https://ir.auriniapharma.com/press-releases/detail/164/aurinia-announces-positive-aurora-phase-3-trial-results.

†https://www.gsk.com/en-gb/media/press-releases/gsk-announces-positive-headline-results-in-phase-3-study-of-benlysta-in-patients-with-lupus-nephritis/.

BICLA, British Isles Combined Lupus Assessment.

First, a successful clinical trial in general SLE was published with anifrolumab, a monoclonal antibody directed at the interferon type 1 receptor.^4 5^ Following a successful phase II trial, an earlier phase III trial of this drug (TULIP 1) had failed as it did not achieve its predefined primary endpoint, the SLE Response Index based on four points (SRI-4).^6^ However, some secondary outcomes in that trial did achieve statistical significance and suggested meaningful improvements with the drug versus placebo. One of these secondary endpoints was the British Isles Combined Lupus Assessment (BICLA). It was then decided to employ this outcome for the TULIP 2 trial and that trial subsequently confirmed efficacy using the BICLA as the primary outcome (in an ironic twist, the TULIP 2 trial also achieved the SRI-4 outcome, so the change in primary outcome, while legitimate before unblinding, turned out not to have been necessary).

Then, in early December, the company Aurinia announced positive results of their phase III clinical trial ‘AURORA’ in lupus nephritis with the calcineurin inhibitor (CNI) voclosporin, a medication related to ciclosporin A and tacrolimus.^1^ The trial has not yet been published or presented, but according to the press release, voclosporin when added to standard of care (SOC) demonstrated a significantly better primary outcome than SOC alone, renal response after 52 weeks, as well as multiple successful secondary outcomes. The efficacy of this medication perhaps did not come as a great surprise, because the class of CNIs have shown suggestions of efficacy in various clinical settings. The innovation in this case lies in the fact that voclosporin lacks the problematic side effects of the older CNIs: there was no increase in deaths, hypertension or worsening renal function in the treated patients.

And next it was announced in a press release that the phase III trial of belimumab in lupus nephritis ‘BLISS-LN’ also achieved its primary endpoint.^2^ Belimumab was approved for use in general SLE almost a decade ago on the basis of two phase III trials,^7 8^ but its efficacy in nephritis had remained unproven, although a post hoc analysis of the subset of patients within those phase III trials had suggested a modest benefit in decreasing proteinuria.^9^ Nevertheless, both for regulatory reasons and to set the minds of treating physicians at ease, it may be of great importance that a positive result now has been obtained. According to the press release, the BLISS-LN trial achieved its primary endpoint showing a statistically significant increase in patients achieving the Primary Efficacy Renal Response over 2 years.

So what are we to make of this unprecedented series of successful phase III clinical trials for lupus? Did the pharmaceutical and biotech companies finally develop effective treatments? Or did the community of lupus scientists, clinical trial experts, regulators and others finally figure out how to do successful trials for SLE in general and lupus nephritis in particular? In fact, both may have been the case. Clearly, a number of unsuccessful clinical trials in lupus failed because the therapy under investigation was truly not or only marginally effective. But other trials were done with agents for which strong and compelling evidence had already been seen, and they ‘failed’ by missing a primary outcome, sometimes by a small margin. An example of the latter category might include the LUNAR trial with rituximab for lupus nephritis, where the difference in non-response favoured rituximab but did not achieve statistical significance,^10^ which could be a case of the trial having been underpowered. This would then be an example of the ‘type 2’ statistical error, failing to prove a difference that really is there. Another example are the two trials of tabalumab, a monoclonal similar to belimumab, that achieved mixed results,^11 12^ and tabalumab was abandoned from further development for what appear to have been commercial reasons as well.

So have we cracked the case? Have we now solved the problem of how to do phase III trials in SLE? Unfortunately, it may be a tad too early to call victory. Two of the recently reported trials were done in lupus nephritis, and these capitalised on extensive investigations that identified optimal renal outcomes in the course of many years. On top of that, with 350–450 patients each, they were large trials, and it may indeed be true that any relevant clinical benefit of a treatment for lupus nephritis can be successfully demonstrated using an appropriate outcome and large numbers of patient. Because of the difficulties of that, the search is on for markers that could predict, early and accurately, which patients are going to do well and which ones are at risk for later renal failure.^13^ The greater challenge has been, and will remain, the population of ‘general’ lupus patients, and the anifrolumab trials, while encouraging, prove a case in point. Before these trials, the only successful drug development programme for SLE had used the SRI-4 outcome, and although several trials using that outcome had failed, until TULIP 1 there was no example of a trial where another outcome performed better: the succes in TULIP 1 of the BICLA despite the failure of SRI-4 is a highly surprising finding, a fact that is further underscored by the observation that both outcomes were successful in TULIP 2. Intensive scrutiny of the full data sets may yet reveal what specifically drove these differences and similarities.

For patients with SLE, the recent string of trial successes may herald a new era of therapeutic options, but here too victory should not be declared too hastily. For those with lupus nephritis, and even though early changes to some extent predict longer term outcomes,^13^ the result that is most relevant for patients—avoidance of renal failure—is measured over much longer time frames than can be tested in randomised trials, so that careful follow-up of the trial cohorts and longitudinal observational studies of other patients will be needed to assign the proper place to novel interventions. And while for patients with general SLE the learnings from the anifrolumab trials may yet translate into improved abilities of doing future trials, it will require some further insights that have so far eluded us. The good news is that many trials are currently underway in SLE with a large variety of medications, including biologicals, kinase inhibitors and other small molecular compounds, and we may yet see the current streak of successful trials extend beyond our wildest dreams—something to hope for in the new decennium!
